# Effect of Intracuff Lidocaine on Postoperative Sore Throat and the Emergence Phenomenon: A Systematic Review and Meta-Analysis of Randomized Controlled Trials

**DOI:** 10.1371/journal.pone.0136184

**Published:** 2015-08-19

**Authors:** Fai Lam, Yu-Cih Lin, Hsiao-Chien Tsai, Ta-Liang Chen, Ka-Wai Tam, Chien-Yu Chen

**Affiliations:** 1 Department of Anesthesiology, Taipei Medical University Hospital, Taipei, Taiwan; 2 Institute of Public Health, College of Medicine, Taipei Medical University, Taipei, Taiwan; 3 Graduate Institute of Nursing, College of Nursing, Taipei Medical University, Taipei, Taiwan; 4 Department of Anesthesiology, School of Medicine, College of Medicine, Taipei Medical University, Taipei, Taiwan; 5 Division of General Surgery, Department of Surgery, Taipei Medical University—Shuang Ho Hospital, New Taipei City, Taiwan; 6 Department of Surgery, School of Medicine, College of Medicine, Taipei Medical University, Taipei, Taiwan; 7 Center for Evidence-based Health Care, Taipei Medical University—Shuang Ho Hospital, New Taipei City, Taiwan; 8 Evidence-based Medicine Center, Taipei Medical University Hospital, Taipei, Taiwan; 9 Graduate Institute of Humanities in Medicine, Taipei Medical University, Taipei, Taiwan; University of Washington, UNITED STATES

## Abstract

**Background:**

Postoperative sore throat and other airway morbidities are common and troublesome after endotracheal tube intubation general anesthesia (ETGA). We propose lidocaine as endotracheal tube (ETT) cuff inflation media to reduce the postintubation-related emergence phenomenon.

**Methods:**

We searched PubMed, EMBASE, and Cochrane databases systematically for randomized controlled trials (RCTs) that have investigated the outcome of intracuff lidocaine versus air or saline in patients receiving ETGA. Using a random-effects model, we conducted a meta-analysis to assess the relative risks (RRs) and mean difference (MD) of the incidence and intensity of relevant adverse outcomes.

**Results:**

We reviewed nineteen trials, which comprised 1566 patients. The incidence of early- and late-phase postoperative sore throat (POST), coughing, agitation, hoarseness, and dysphonia decreased significantly in lidocaine groups, with RRs of 0.46 (95% confidence interval [CI]: 0.31 to 0.68), 0.41 (95% CI: 0.25 to 0.66), 0.43 (95% CI: 0.31 to 0.62), 0.37 (95% CI: 0.25 to 0.55), 0.43 (95% CI: 0.29 to 0.63), and 0.19 (95% CI: 0.08 to 0.5), respectively, when compared with the control groups. The severity of POST also reduced significantly (mean difference [MD] -16.43 mm, 95% CI: -21.48 to -11.38) at 1 h and (MD -10.22 mm, 95% CI: -13.5 to -6.94) at 24 h. Both alkalinized and non-alkalinized lidocaine in the subgroup analyses showed significant benefits in emergence phenomena prevention compared with the control.

**Conclusion:**

Our results indicate that both alkalinized and non-alkalinized intracuff lidocaine may prevent and alleviate POST and postintubation-related emergence phenomena.

## Introduction

The postintubation-related emergence phenomenon is a cluster of airway complications associated with tracheal intubation or extubation after general anesthesia. Various symptoms result from mucosal injury or inflammation caused by airway instrumentation (ie, laryngoscope and suctioning) or the irritating effects of a foreign object (ie, endotracheal tube (ETT)) [1]. Postoperative sore throat (POST) is one of the most undesirable morbidities that occurs in approximately 50% or more surgical patients [2–6]. During emergence from general anesthesia, patients may experience vigorous coughing, agitation or restlessness which might increase intracranial, intra-thoracic or intra-abdominal pressure, resulting in bronchospasm, wound dehiscence, and bleeding [7–9]. Other laryngeal complication such as hoarseness, dysphonia, or dysphagia was also noted during the postoperative care [10–12].

Prevention strategies for POST and other airway complications during emergence have recently shifted from non-pharmacological (e.g., ETT size, cuff pressure or volume control) to pharmacological strategies [13]. Various prophylactic interventions such as anti-inflammatory drugs, opioids, steroids, or local anesthetics have been employed extensively [14–18]. Lidocaine is one of the most commonly used drugs for preventing POST, and its efficacy was evaluated in a Cochrane review in 2009 [19]. Nevertheless, the clinical application of the results of this review may still be equivocal, because the route of lidocaine administration was not adequately confined, and its effectiveness on other relevant morbidities was not fully considered.

Lidocaine, when administered as a cuff inflation medium, may protect the tracheal mucosa through its continuous topical anesthetic effect, and prevent the diffusion of nitrous oxide into the cuff [20–22]. Alkalinized lidocaine has an advantage over its non-alkalinized variety, with a quicker onset, duration, and quality of the block [10–12]. Several randomized controlled trials (RCTs) have investigated the prophylactic efficacy of intracuff lidocaine on the postintubation-related emergence phenomenon, but the results remain inconclusive [12, 22–24]. Thus, we conducted a systematic review and a meta-analysis of the evidence available to date regarding patient outcomes where alkalinized or non-alkalinized lidocaine was administered as a cuff medium of an ETT for patients undergoing general anesthesia.

## Materials and Methods

We conducted a meta-analysis of RCTs to evaluate the preventive effect of POST and other postintubation-related emergence phenomena involving intracuff lidocaine compared with air or saline, in accordance with the PRISMA guideline [25]. A review protocol was written prior to conducting the study and registered (PROSPERO registration number: CRD42014010819).

### Inclusion and Exclusion Criteria

Two reviewers (Lam and Lin) screened all articles and abstracts independently and jointly for the following inclusion criteria: The study (1) was an RCT; (2) was an evaluation of intracuff lidocaine outcome in patients undergoing endotracheal tube intubation general anesthesia (ETGA); and (3) included any outcome of interest (the incidence and severity of any airway complication during emergence). We excluded previous RCTs from the meta-analysis based on the following criteria: (1) emergency operation; (2) small-scale preliminary pilot study; (3) the appropriate data could not be extracted or calculated from the published results; or (4) the study conducted a duplicate reporting of patient cohorts.

### Search Strategy and Study Selection

We performed a comprehensive literature search in several databases, including PubMed, EMBASE, Google Scholar, the Cochrane central registers of controlled trial databases, and the ClinicalTrials.gov registry (http://clinicaltrials.gov/). We used free text and MeSH terms individually, and in various combinations. We used the following keywords for the medical subject heading and free text searches: *emergence phenomenon*, *postoperative sore throat*, *cough*, *hoarseness*, *pharyngitis*, *dysphonia*, *dysphagia*, *bronchial spasm*, *laryngospasm*, *difficult swallowing*; *lidocaine* OR *lignocaine* OR *xylocaine*; and *endotracheal tube* OR *intubation* OR *extubation* (S1 Table). We used related citations in the PubMed search tool to broaden each search, and we reviewed all abstracts, study reports, and related citations retrieved. No language restrictions were imposed. The last search was performed in February 2015.

### Data Extraction

Two reviewers (Lam and Lin) independently extracted the baseline and outcome data, including the study design, participant information, the inclusion and exclusion criteria, the anesthetic techniques used, the airway devices employed, the type of surgery, and any resulting complications. A third reviewer (CY Chen) resolved any inconsistencies between the findings of the 2 reviewers.

### Methodological Quality Appraisal

We assessed the methodological quality of each trial based on the Cochrane risk of bias table [26], which includes the adequacy of randomization, the allocation concealment, the blinding of patients and outcome assessors, the length of follow-up, the reporting of study withdrawals, the performance of an intention-to-treat analysis, and other possible sources of bias.

### Outcome Measures and Statistical Analysis

The primary outcomes were the incidence and severity of POST within 24 h postoperation. The severity of POST was standardized and synthesized using pain scores (the visual analog scale or numeric rating scale) from 0 (*no pain*) to 100 mm (*worst pain*) at 1 h and 24 h after surgery. The secondary outcome included the incidence of other airway morbidities during emergence, such as coughing, agitation or restlessness, hoarseness, dysphagia, dysphonia, and desaturation. The control group included either patients with intracuff air or who inflated saline. Any amount or concentration of sodium bicarbonate (NaHCO_3_) added to the lidocaine solution was referred to as being in the alkalinized subgroup.

We entered all data and analyzed them using Review Manager, version 5.3 (Cochrane Collaboration, Oxford, England). When necessary, we estimated standard deviations from the confidence interval (CI) limits, the standard error, or the range values provided in the past studies. We reported the effect sizes of dichotomous outcomes as risks ratios (RR), and the mean difference (MD) was reported for continuous outcomes. The precision of the effect sizes was based on a 95% CI. A pooled estimate of the RR was computed using the DerSimonian and Laird random-effects model [27]. This model provides an appropriate estimate of the average treatment effect when trials are statistically heterogeneous, and it typically yields relatively wide CIs, resulting in a more conservative statistical claim. To evaluate the statistical heterogeneity and any inconsistencies in the treatment effects across studies, we used the Cochrane Q test and *I* ^2^ statistics, respectively. Statistical significance was set at .10 for the Cochrane Q tests. The proportion of the total outcome variability attributable to the variability across studies was quantified as *I* ^*2*^. We conducted sensitivity analyses to assess any impact of the study quality on the effect estimates. Subgroup analyses were also performed by pooling estimates for similar subsets of patients across trials, where available. We assessed the publication bias by using a funnel plot to determine whether a bias exists toward the publication of studies with positive results among studies with a smaller sample.

## Results

### Study Selection and Characteristics

**Fig 1:**
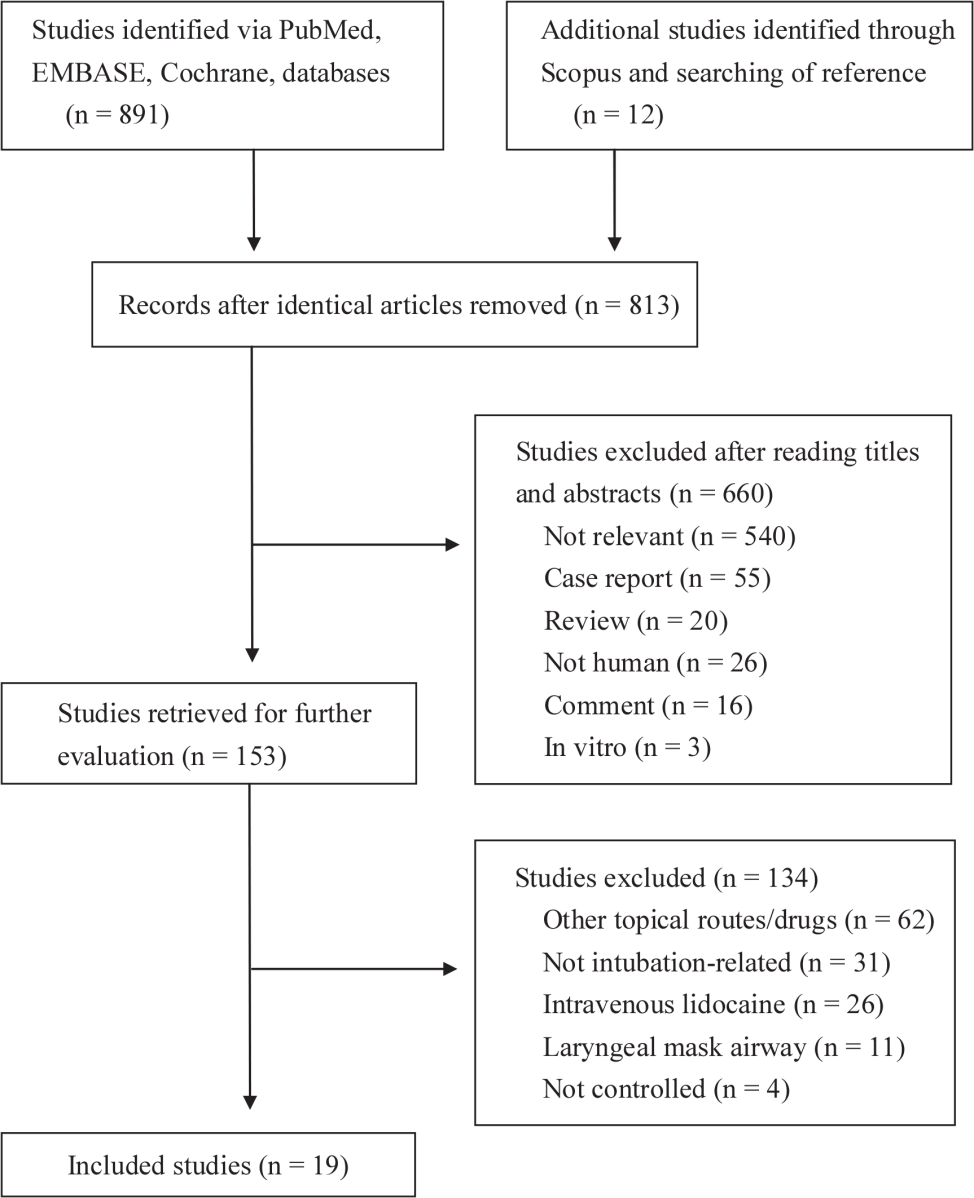
A flowchart showing the selection of the randomized controlled trials for our meta-analysis.

Nineteen RCTs, comprising 1566 participants, met the inclusion criteria. The flowchart in Fig 1 shows the process for screening and including RCTs. Our initial search yielded 813 citations. Based on the screening criteria for titles and abstracts, we excluded 660 studies. After reviewing the full text of the remaining 153 reports, we found that 19 eligible RCTs published between 1997 and 2014 met our inclusion criteria [10–12, 22–24, 28–40]. Among the selected studies, 18 were published in English, and one in Spanish [40]; 12 investigated the effect of intracuff lidocaine without alkalinization [10, 22, 23, 28–33, 38–40], whereas 9 focused on alkalinized lidocaine [10–12, 24, 33–37]; and 8 used an inflated ETT cuff with saline as the control [24, 28, 29, 31, 33, 36, 39, 40]; 6 chose air instead [10–12, 22, 35, 38]; and both media were investigated 5 trials [23, 30, 32, 34, 37]. In certain RCTs, the researchers have simultaneously compared the efficacy of intracuff lidocaine with other routes or interventions, such as a direct larynx spray under a laryngoscope [24, 31, 38, 40], the application of an ETT lubricant [10–12, 34, 36, 38], intravenous injection [38, 40], and intracuff lidocaine under different temperatures [33] and concentrations [29]. The substantial inconsistencies between the anesthetic techniques employed included the implementation of premedication [11, 12, 28, 30, 32, 34–36], N_2_O [10, 11, 22, 23, 29, 30, 32–39], total intravenous anesthetics [31, 37], continuous opioid administration [12, 31, 32, 35, 36, 40], and the use of the minimal leakage technique (MLT) for cuff inflation [10–12, 22–24, 28–30, 32, 34–38]. The samples of the selected trials numbered from 38 to 204 patients. More detailed information on the patient characteristics, anesthetic techniques, surgical procedures, and the interventions adopted are listed in Table 1.

**Table 1:** The Characteristics of the Selected Randomized Controlled Trials.

First Author, Year	ETT Size (M/F)/ Intubator	Surgery/ ASA Status	Anesthetic Technique	Patient Number (male %)	Intervention
Ahmady, 2013^28^	3 + age/4 mm/ VS	Dental/ I-II	Induced by fentanyl 2 μg kg^-1^, propofol 2.5 mg kg^-1^, rocuronium 0.5 mg kg^-1^; maintained by 50% O_2_, 2–3% sevoflurane, fentanyl 1–2 μg kg^-1^	La:25 (64)	La: 1.5 mL 2% lidocaine + 1.5 mL 8.4% NaHCO_3_ by MLT
				Cs:25 (60)	Cs: 3 mL saline by MLT
Altintas, 2000^29^	8/7 mm/ unclear	PS/ I-II	Induced by fentanyl 2 μg kg^-1^, propofol 2 mg kg^-1^, atracurium 0.5 mg kg^-1^; maintained by 50% N_2_O, 1–2% isoflurane, fentanyl 1 μg kg^-1^	Ln: 36 (42)	Ln: 10% lidocaine in cuff by MLT (< 5 ml)
				Cs: 34 (47)	Cs: saline in cuff by MLT
Bajaj, 2004^30^	Unclear/ unclear	Elective/ I-II	Induced by thiopentone, suxamethonium chloride 2 mg kg^-1^; maintained by 60% N_2_O, halothane, vecuronium 0.08–0.1 mg kg^-1^ or atracurium 0.5 mg kg^-1^	Ln: 20	Ln: 4% lidocaine in cuff by MLT & PREFILL
				Ca: 20	Ca: air in cuff by MLT
				Cs: 20	Cs: saline in cuff by MLT
				N60: 20	N60: 60% N_2_O with 40% O_2_ in cuff by MLT
Bousselmi, 2014^31^	7.5/7 mm/ unclear	Elective/ I-III	Induced by propofol 2.5 mg kg^-1^, remifentanil 0.5 μg kg^-1^, cisatracurium 0.15 mg kg^-1^; maintained by continuous infusion of propofol and remifentanil, bolus cisatracurium	Ln:20 (65)	Ln: 4 mL 2% lidocaine in cuff; 4 mL saline on LARYNX
				Ls:20 (55)	Ls: 4 mL saline in cuff; 4 mL 2% lidocaine on LARYNX
				Ls*:20 (70)	Ls*: 4 mL 2% lidocaine in cuff; 4 mL 2% lidocaine on LARYNX
				Cs: 20 (55)	Cs: 4 mL saline in cuff; 4 mL saline on LARYNX
D’ Aragon, 2013^24^	7 mm/ VS or senior R	GYN/ I-II	Induced by fentanyl 2–3 μg kg^-1^, propofol 2–3 mg kg^-1^, rocuronium 0.6 mg kg^-1^; maintained by 50% O_2_, desflurane, fentanyl 1 μg kg^-1^, rocuronium 0.15 mg kg^-1^	La: 30 (0)	La: 2 mL 2% lidocaine in cuff + 8.4% NaHCO_3_ until 30 cmH_2_O; saline on LARYNX
				Cs: 29 (0)	Cs: saline in cuff until 30 cmH_2_O; saline on LARYNX
				Ls: 29 (0)	Ls: saline in cuff until 30 cmH_2_O; 4 mL 4% lidocaine on LARYNX
				Ls*: 28 (0)	Ls*: 2 mL 2% lidocaine in cuff + 8.4% NaHCO_3_ until 30 cmH_2_O; 4 mL 4% lidocaine on LARYNX
Estebe, 2002^10^	Unclear/ unclear	L-spine/ I-III	Maintained by 70% N_2_O, isoflurane, sufentanil, rocuronium	Ln: 25 (52)	Ln: 2 mL 2% lidocaine in cuff + water by MLT + 2 mL water; water on cuff
				La: 25 (52)	La: 2 mL 2% lidocaine in cuff + 8.4% NaHCO_3_ by MLT + 2 mL 8.4% NaHCO_3_; water on cuff
				Ca: 25 (56)	Ca: air in cuff by MLT + 2 mL air; water on cuff
Estebe, 2004^11^	7–7.5/6.5–7 mm/ unclear	L-spine/ I-III	Induced by thiopental 4–6 mg kg^-1^, sufentanil 0.5 μg kg^-1^, rocuronium 0.5 mg kg^-1^; maintained by 70% N_2_O, isofluarne, sufentanil, rocuronium	La: 20 (60)	La: 2 mL 2% lidocaine in cuff + 8.4% NaHCO_3_ by MLT + 2 mL 8.4% NaHCO_3_; water on cuff
				Ca: 20 (65)	Ca: air in cuff by MLT + 2 mL air; water on cuff
				Lg: 20 (70)	Lg: 2 mL 2% lidocaine in cuff + 8.4% NaHCO_3_ by MLT + 2 mL 8.4% NaHCO_3_; water-soluble gel on cuff
Estebe, 2005^12^	7–7.5/6.5–7 mm/ unclear	Thyroidect-omy/ I-II	Induced by propofol 2.5 mg kg^-1^, sufentanil 0.35 μg kg^-1^ min^-1^, atracurium 0.6 mg kg^-1^; maintained by 50% O_2_, 50% air, 1–1.2% sevoflurane, sufentanil 0.35 μg kg^-1^ min^-1^	La: 20 (25)	La: 2 mL 2% lidocaine in cuff + 8.4% NaHCO_3_ by MLT + 1 mL 8.4% NaHCO_3_; water on cuff
				La*: 20 (25)	La*: 2 mL 2% lidocaine in cuff + 1.4% NaHCO_3_ by MLT + 1 mL 1.4% NaHCO_3_; water on cuff
				Ca: 20 (15)	Ca: air in cuff by MLT + 1 mL air; water on cuff
Fragan, 2000^32^	8.5/7.5 mm/ unclear	Ortho, PS, Uro, GS/ I-II	Induced by fentanyl 1.5 μg kg^-1^, propofol 2.5 mg kg^-1^, vecuronium 0.1 mg kg^-1^; maintained by 65% N_2_O, 1.2–1.5% isoflurane, fentanyl 1–1.5 μg kg^-1^ min^-1^	Ln: 18	Ln: 4% lidocaine in cuff by MLTCa: air in cuff by MLT
				Cs: 18	Cs: saline in cuff by MLT
				Ca: 21	Ca: air in cuff by MLT
Huang, 1998^33^	7–8.5 mm/ unclear	Elective/ I-II	Induced by atropine 0.4 mg, fentanyl 2 μg kg^-1^, atracurium 5 mg, thiopental 3–5 mg kg^-1^, succinylcholine 1.5 mg kg^-1^; maintained by N_2_O, enflurane, atracurium	Ln: 20 (50)	Ln: 6 mL 4% lidocaine in cuff
				La: 20 (45)	La: 5 mL 4% lidocaine + 1 mL 7% NaHCO_3_ in cuff
				La: 20 (60)	La°: 38°C 5 mL 4% lidocaine + 1 mL 7% NaHCO_3_ in cuff
				Cs: 20 (55)	Cs: 6 mL saline in cuff
Jaichandran, 2009^34^	8–8.5/7-7.5 mm/ unclear	Oph/I-II	Induced by propofol 1.5 mg kg^-1^, vecuronium 0.1 mg kg^-1^, maintained by 70% N_2_O, 0.6% isoflurane, vecuronium	La: 25 (80)	La: 6 mL 2% lidocaine + 0.5 mL 7.5% NaHCO_3_ in cuff by MLT; water-soluble gel on cuff
				Ca: 25 (80)	Ca: air in cuff by MLT; water-soluble gel on cuff
				Cs: 25 (84)	Cs: 6 mL saline in cuff by MLT; water-soluble gel on cuff
Navarro, 1997^22^	7.5/7 mm/ unclear	Elective/ I-II	Induced by thiopental 3–6 mg kg^-1^ or propofol 2–2.5 mg kg^-1^, succinylcholine 1.5 mg kg^-1^; maintained by 65% N_2_O, isoflurane, opioid, NMBA	Ln: 53 (15)	Ln: 8 mL 4% lidocaine in cuff by MLT & PREFILL
				Ca: 53 (19)	Ca: air in cuff by MLT
Navarro, 2007^35^	7.5 mm/ VS	GYN, PS/ I-II	Induced by propofol 2 mg kg^-1^, sufentanil 0.7 mg kg^-1^, rocuronium 0.6 mg kg^-1^; maintained by 65% N_2_O, isoflurane, rocuronium, sufentanil	La: 25	La: 2% lidocaine + 8.4% NaHCO_3_ in cuff until 20 cmH_2_O
				Ca: 25	Ca: air in cuff until 20 cmH_2_O
Navarro, 2012^36^	8/7.5 mm/ unclear	GYN, PS, Ortho/ I-II	Maintained by balanced anesthesia with 60% N_2_O, isoflurane, sufentanil, rocuronium infusion	La: 25 (0)	La: alkalinized lidocaine (2% lidocaine: 8.4% NaHCO_3_ = 19:1) in cuff by MLT; 4 mL water-soluble gel on cuff
				Cs: 25 (0)	Cs: saline in cuff by MLT; 4 mL water-soluble gel on cuff
Porter, 1999^23^	6.5–7 mm/ unclear	GYN/ I-III	Induced by propofol, fentanyl, mivacurium or rocuronium or vecuronium; maintained by volatile inhalation agents with or without N_2_O	Ln: 26 (0)	Ln: 2% lidocaine in cuff by MLT
				Ca: 24 (0)	Ca: air in cuff by MLT
				Cs: 25 (0)	Cs: saline in cuff by MLT
Shroff, 2009^37^	Unclear/ unclear	Elective/ I-II	Balanced anesthesia, 60% N_2_O, opioid, propofol, benzodiazepine, NMBA	La: 50 (32)	La: 2 mL 2% lidocaine in cuff + 7.5% NaHCO_3_ by MLT
				Ca: 50 (38)	Ca: air in cuff by MLT
				Cs: 50 (32)	Cs: saline in cuff by MLT
Soltani, 2002^38^	8–8.5/7-7.5 mm/ unclear	Cataract/ I-II	Induced by lidocaine 1.5 mg kg^-1^, alfentanil 10 μg kg^-1^, thiopental 5 mg kg^-1^, gallamine 20 mg, succinylcholine 1.5 mg kg^-1^; maintained by 50% N_2_O, 1–2% halothane	Ln: 34	Ln: 7–8 mL 2% lidocaine in cuff by MLT & PREFILL
				Ca: 34	Ca: air in cuff by MLT; saline on cuff
				Ls: 34	Ls: 3 puffs 10% lidocaine on LARYNX
				Ls^#^: 34	Ls^#^: 3 puffs 10% lidocaine on cuff
				Lj: 34	Lj: 2.5 g of 2% lidocaine jelly on cuff
				Lv: 34	Lv: 1.5 mg kg^-1^ intravenous lidocaine at the end of surgery
Wetzel, 2008^39^	Unclear/ unclear	Elective/ I-III	Maintained by N_2_O, volatile inhalation agents	Ln:19 (21)	Ln: 5 mL 4% lidocaine in cuff
				Cs:19 (32)	Cs: 5 mL saline in cuff
Zamora, 2007^40^	8/7-7.5 mm/ unclear	GS, Oph, PS, Ortho, Uro, GYN/ I-II	Induced by fentanyl 2 μg kg^-1^, propofol 2.5 mg kg^-1^, rocuronium 0.6 mg kg^-1^; maintained by 100% O_2_, 2% sevoflurane, rocuronium, fentanyl 3–4 μg kg^-1^ min^-1^,	Ln: 19 (53)	Ln: 5 mL 2% lidocaine in cuff; 5 mL saline on LARYNXv: 5 mL 2% intravenous lidocaine before intubation; 5 mL saline on LARYNX
				Cs: 20 (60)	Cs: 5 mL saline in cuff; 5 mL saline on LARYNX
				Ls: 19 (47	Ls: 5 mL intravenous saline before intubation; 5 mL 2% lidocaine on LARYNX
				Lv: 20 (35)	Lv: 5 mL 2% intravenous lidocaine before intubation; 5 mL saline on LARYNX

Ca = control group, cuff injected with air; Cs = control group, cuff injected with saline; Dental = dental surgery; GS = general surgery; GYN = gynecologic surgery; L-spine = lumbar spine surgery; La = cuff injected with alkalinized lidocaine; La* = cuff injected with less alkalinized lidocaine; La° = cuff injected with alkalinized lidocaine at 38°C; LARYNX = media sprayed on larynx under laryngoscope; Lg = cuff lubricated with water-soluble gel; Lj = cuff lubricated with lidocaine jelly; Ln = cuff injected with non-alkalinized lidocaine; Ls = lidocaine sprayed on LARYNX; Ls* = lidocaine sprayed on LARYNX and cuff injected with lidocaine; Ls^#^ = lidocaine sprayed on cuff; Lv = intravenous lidocaine injection; MLT = minimal leakage technique; N60 = cuff injected with 60% N_2_O; NMBA = Neuromuscular blocking agents; Oph = ophthalmic surgery; Ortho = orthopedic surgery; PS = plastic surgery; PREFILL = cuff injected with lidocaine 90 min before intubation; R = resident; Uro = urologic surgery; VS = certified anesthesiologist

Our assessment of the methodological quality of the 19 selected studies is listed in Table 2. Seven studies had described the methods of allocation generation [12, 22–24, 31, 38, 40]; 3 studies had detailed the methods of allocation concealment [28, 37, 40]; and detailed information regarding the blinding of patients as well as assessors has been specified in 11 studies [10, 12, 24, 28, 31, 33, 36–40]. Sixteen studies had performed an intention-to-treat analysis [10–12, 22, 23, 28–31, 33–39]. Other biases and limitations included the lack of disclosure in sex percentage [30, 32, 36, 38], ETT size [10, 30, 37, 39], intubator [10–12, 22, 23, 29–34, 36–40], surgical type [22, 30, 31, 33, 37, 39], clear definition of POST incidence [11–12, 22–24, 28–33, 35–40], the anesthesia method [39], and participant restrictions included only being female [23, 24, 35], a smoker [36, 39], and pediatrics [28].

**Table 2:** The Methodological Quality Assessment of Selected Trials.

First Author, Year	Country	Allocation Generation	Allocation Concealment	Blinding	Loss of Follow-up	Data Analysis	Other Bias and Limitations
Ahmady, 2013^28^	Saudi Arabia	Unclear	Sealed envelopes	Double	0	ITT	Child only
Altintas, 2000^29^	Turkey	Unclear	Unclear	Assessor	0	ITT	High concentration lidocaine (10%)
Bajaj, 2004^30^	India	Unclear	Unclear	Assessor	0	ITT	No gender, surgical type and ETT size reported
Bousselmi, 2014^31^	Tunisia	Software	Unclear	Double	0	ITT	No surgical type reported
D’ Aragon, 2013^24^	Canada	Permuted block	Unclear	Double	3.3%	PP	Female only
Estebe, 2002^10^	France	Unclear	Unclear	Double	0	ITT	No ETT size reported
Estebe, 2004^11^	France	Unclear	Unclear	Assessor	0	ITT	
Estebe, 2005^12^	France	Computerized list	Unclear	Double	0	ITT	Surgical site at neck
Fragan, 2000^32^	Ireland	Unclear	Unclear	Assessor	10%	PP	No gender reported
Huang, 1998^33^	Taiwan	Unclear	Unclear	Double	0	ITT	No surgical type reported
Jaichandran, 2009^34^	India	Unclear	Unclear	Assessor	0	ITT	Surgical time < 90 min
Navarro, 1997^22^	USA	Random number table	Unclear	Assessor	0	ITT	No surgical type reported
Navarro, 2007^35^	Brazil	Unclear	Unclear	Assessor	0	ITT	Female only
Navarro, 2012^36^	Brazil	Unclear	Unclear	Double	0	ITT	Smoker only, no gender reported
Porter, 1999^23^	USA	Random number table	Unclear	Unclear	0	ITT	Female only
Shroff, 2009^37^	India	Unclear	Sealed envelopes	Double	0	ITT	No ETT size & surgical type reported
Soltani, 2002^38^	Iran	Convenience sampling	Unclear	Double	0	ITT	No gender reported
Wetzel, 2008^39^	USA	Unclear	Unclear	Double	0	ITT	Smoker only; no ETT size, surgical type and anesthesia reported
Zamora, 2007^40^	Mexico	Random number table	Sealed envelopes	Double	2.5%	PP	

ETT = endotracheal tube; ITT = intention-to-treat; PP = per-protocol.

### Incidence and Severity of POST

**Fig 2:**
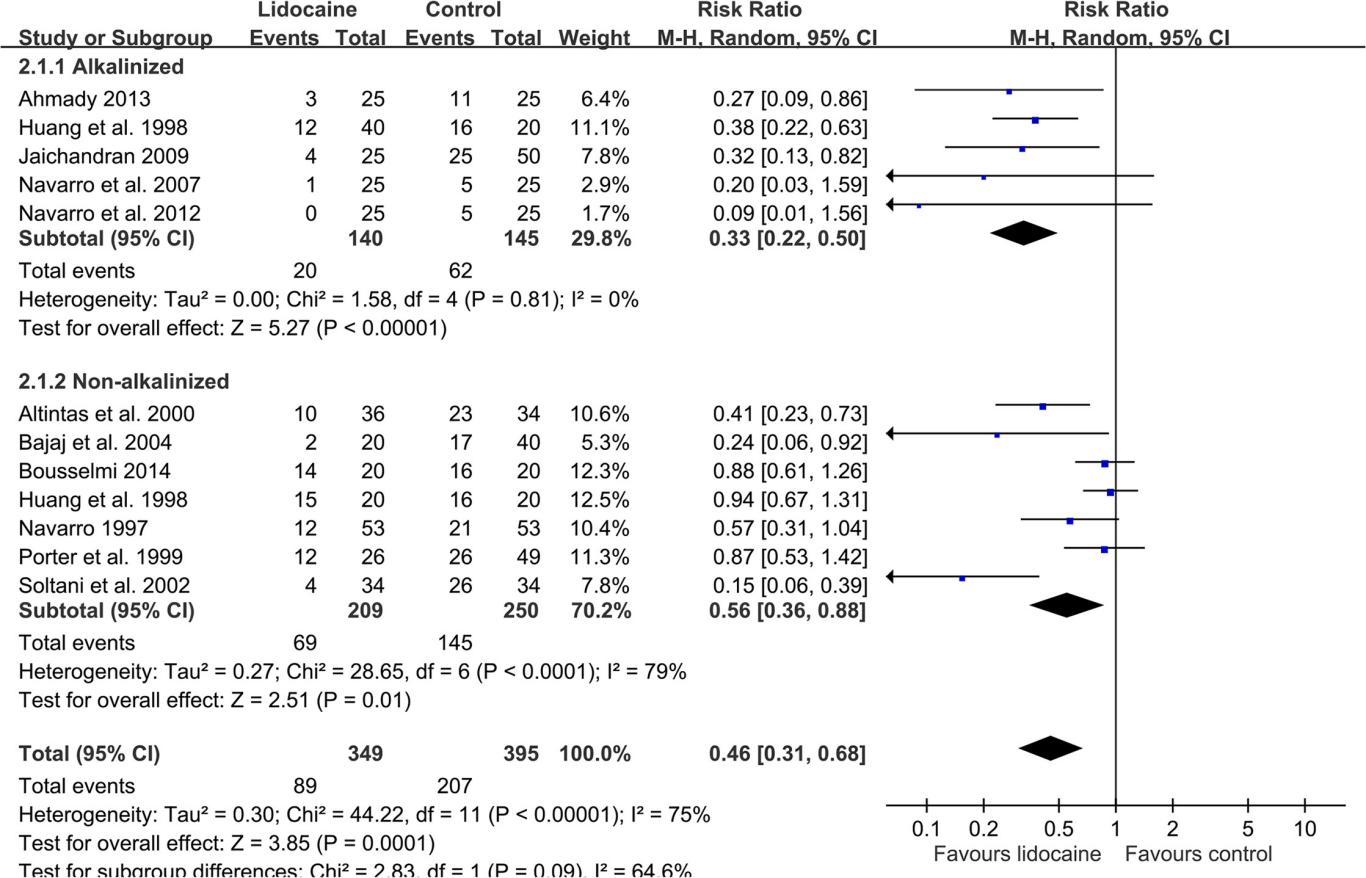
A forest plot showing a comparison of intracuff lidocaine (alkalinized and non-alkalinized) used and the control groups, as well as the incidence of POST at 1 h.

**Fig 3:**
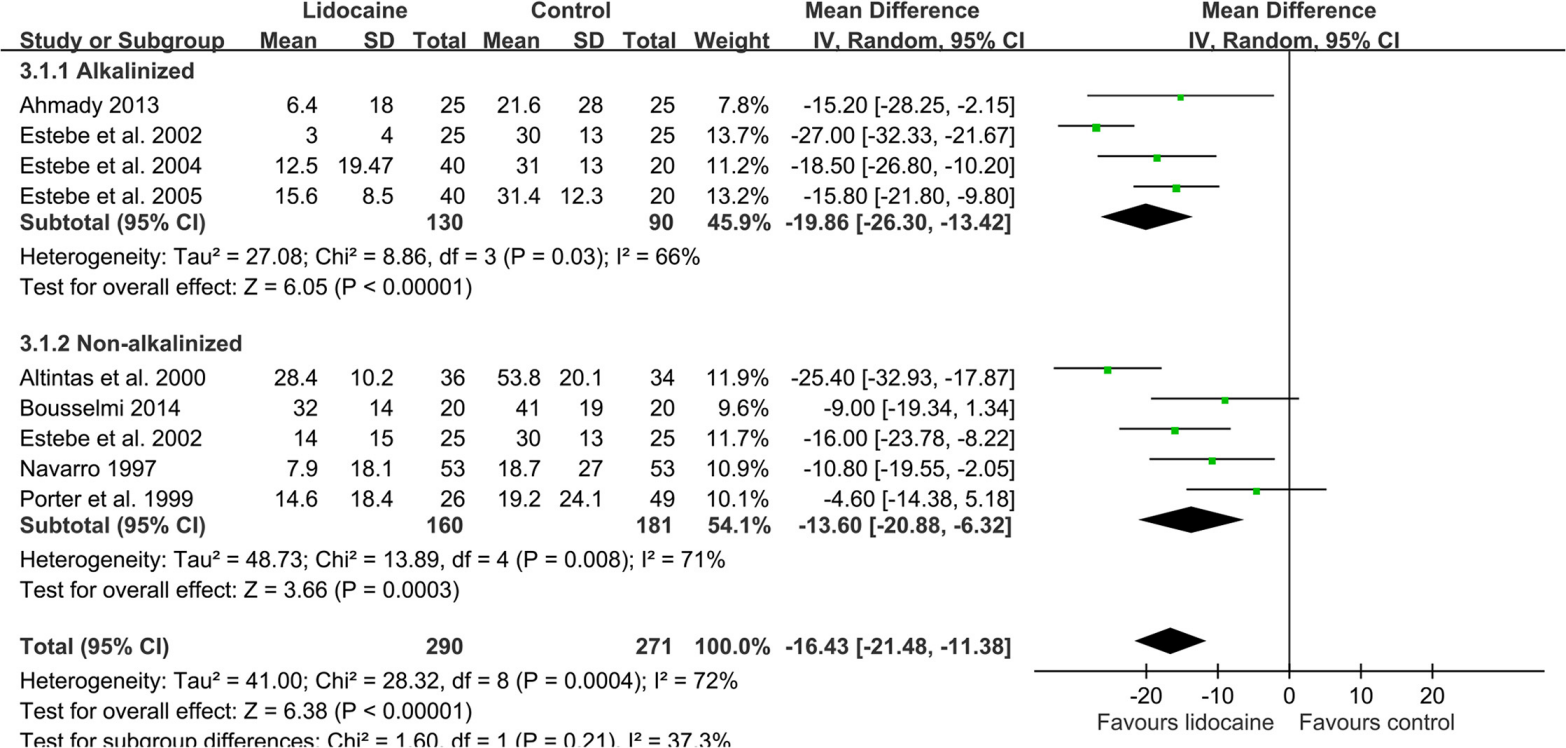
The effect of intracuff lidocaine (alkalinized and non-alkalinized) used on the POST pain score at 1 h.

Eleven studies investigated the incidence of POST. In these studies, only Jaichandran et al. [34] clearly defined POST incidence as VAS greater or equal to 10 mm, whereas Estebe et al [10] by binary questions. Others did not mention their cut-off value or criteria of POST. The aggregate effect of the 11 studies (n = 744) having evaluated the effect of intracuff lidocaine on the incidence of early-phase POST have favored lidocaine over the control (RR 0.46, 95% CI: 0.31 to 0.68; Fig 2) at 1 h [22, 23, 28–31, 33–36, 38]. Subgroup analysis revealed that both alkalinized lidocaine [28, 33–36] (RR 0.33, 95% CI: 0.22 to 0.50) and non-alkalinized lidocaine [22, 23, 29–31, 33, 38] (RR 0.56, 95% CI: 0.36 to 0.88) offered protection compared with control groups. Regarding the pain intensity of POST at 1 h, the mean in intervention group was 14.1 mm while the control was 29.1 mm; the mean difference between lidocaine and the control was significant (-16.43 mm, 95% CI: -21.48 to -11.38) (Fig 3), both in the alkalinized [10–12, 28] (-19.86 mm, 95% CI: -26.3 to -13.42) and non-alkalinized [10, 22, 23, 29, 31] (-13.6 mm, 95% CI: -20.88 to -6.32) subgroups.

**Fig 4:**
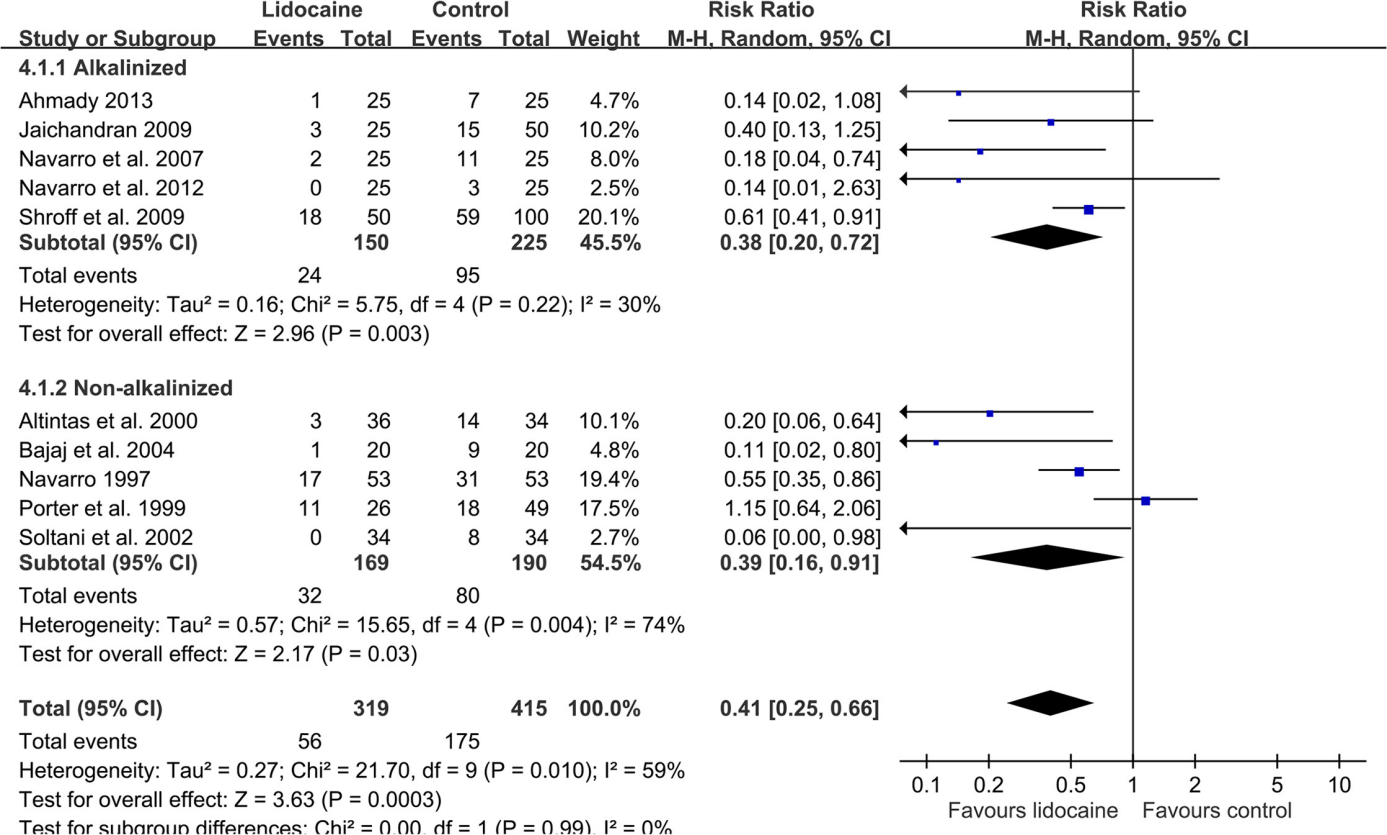
A forest plot showing a comparison of intracuff lidocaine (alkalinized and non-alkalinized) used and the control groups, as well as the incidence of POST at 24 h.

**Fig 5:**
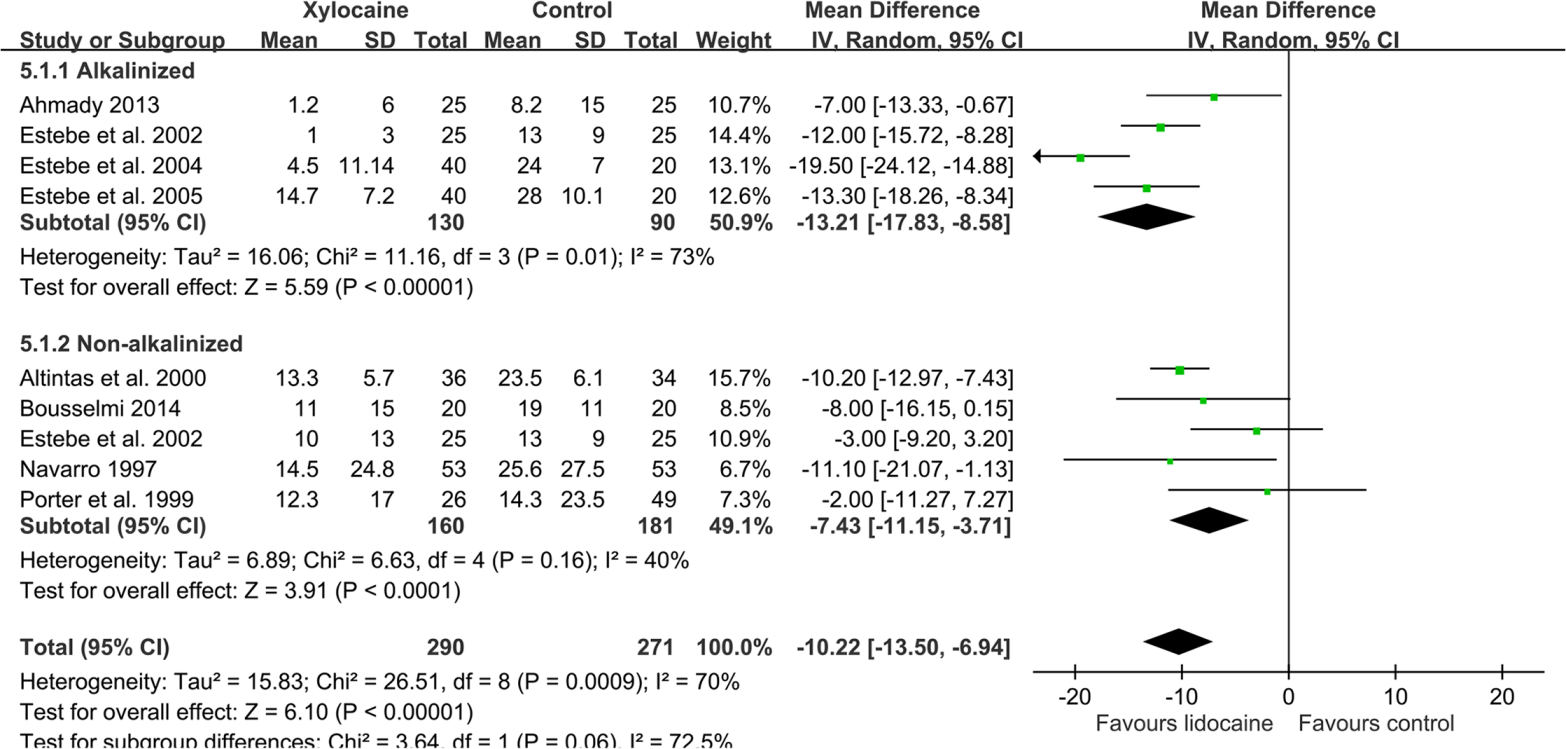
The effect of intracuff lidocaine (alkalinized and non-alkalinized) used on the POST pain score at 24 h.

In the 10 studies (n = 734) that evaluated intracuff lidocaine on the specific incidence of late-phase POST at 24 h, a significant benefit of lidocaine compared with the control was identified (RR 0.41, 95%: CI 0.25 to 0.66; Fig 4) [22, 23, 28–30, 34–38]. Subgroup analysis did not demonstrate any effect of lidocaine alkalinization on this outcome [28, 34–37]. Although the severity of POST at 24 h was generally reduced in both groups (9.8 mm in intervention groups versus 17.3 mm in control groups), intracuff lidocaine still offers a significant protective effect compared with the control (MD -10.22 mm; 95% CI, -13.5 to -6.94; Fig 5) [10–12, 22, 23, 28, 29, 31]. Both the alkalinized [10–12, 28] (MD -13.21 mm; 95% CI, -17.83 to -8.58) and non-alkalinized [10, 22, 23, 29, 31] (MD -7.43 mm; 95% CI, -11.15 to -8.58) subgroups had a significantly lower pain score.

For an evaluation of the publication bias, we plotted the incidence of POST in the lidocaine and control groups against precision groups by using a funnel plot. The funnel plot indicated a missing limb, revealing a potential for publication bias (S1 Fig).

### Incidence of Other Morbidities during Emergence

The meta-analysis of other emergence phenomena listed in Table 3 indicated that the incidences of coughing [10–12, 23, 24, 28, 29, 31, 32, 34, 36, 37, 40], agitation or restlessness [10–12, 24, 35, 37], hoarseness [10–12, 28,30, 35–37], and dysphonia [10–12, 31] decreased significantly in the overall lidocaine groups, with RRs of 0.43 (95% CI: 0.31 to 0.62), 0.37 (95% CI: 0.25 to 0.55), 0.43 (95% CI: 0.29 to 0.63), and 0.19 (95% CI: 0.08 to 0.5), respectively, compared with the control groups. Overall lidocaine provided significant protection in the occurrences of coughing [10–12, 23, 24, 28, 29, 31, 32, 34, 36, 37, 40], agitation or restlessness [10–12, 24, 35, 37], hoarseness [10–12, 28, 30, 35–37], and dysphonia [10–12, 31], with RRs of 0.43 (95% CI: 0.31 to 0.62), 0.37 (95% CI: 0.25 to 0.55), 0.43 (95% CI: 0.29 to 0.63), and 0.19 (95% CI: 0.08 to 0.5), respectively, compared with the control groups. The overall effect of 6 studies (n = 339) evaluating intracuff lidocaine on postoperative dysphagia did not show a significant benefit of lidocaine (RR 0.73, 95% CI: 0.23 to 2.32) [10–12, 24, 30, 31].

**Table 3:** Intracuff Lidocaine for Preventing Other Complications.

Emergence Phenomenon: Subgroup	Number of Studies	Number of Patients	Risk Ratio (95% CI)	*I* ^2^	*P* value
Coughing:					
Overall	13^10−12,23,24,28,29,31,32,34,36,37,40^	885	0.43 (0.31 to 0.62)	85%	<0.01
Alkalinized	8^10−12,24,28,34,36,37^	554	0.39 (0.25 to 0.6)	79%	<0.01
Non-alkalinized	6^10,29,31–33,40^	331	0.51 (0.29 to 0.9)	86%	0.02
Agitation or restlessness:					
Overall	6^10−12,24,35,37^	479	0.37 (0.25 to 0.55)	5%	<0.01
Alkalinized	6^10−12,24,35,37^	429	0.36 (0.23 to 0.58)	21%	<0.01
Non-alkalinized	1^10^	50	0.38 (0.11 to 1.25)	NA	0.11
Hoarseness:					
Overall	8^10−12,28,30,35–37^	580	0.43 (0.29 to 0.63)	56%	<0.01
Alkalinized	6^10−12,28,35,36^	320	0.39 (0.2 to 0.73)	69%	<0.01
Non-alkalinized	3^10,30,37^	260	0.48 (0.34 to 0.68)	0%	<0.01
Dysphonia:					
Overall	4^10−12,31^	260	0.19 (0.08 to 0.5)	0%	<0.01
Alkalinized	3^10−12^	170	0.21 (0.05 to 0.84)	25%	0.03
Non-alkalinized	2^10,31^	90	0.18 (0.03 to 1.0)	0%	0.05
Dysphagia:					
Overall	6^10−12,24,30,31^	339	0.73 (0.23 to 2.32)	60%	0.59
Alkalinized	4^10−12,24^	189	1.93 (0.53 to 7.01)	NA	0.32
Non-alkalinized	3^10,30,31^	150	0.46 (0.13 to 1.57)	48%	0.17

NA = Not applicable

Other rare complications have also been assessed and reported carefully, such as cuff rupture [10–12, 33, 36, 38], laryngospasm [10–12, 24, 28, 29, 32, 37, 38], stridor [28, 30, 37, 38], and cyanosis or desaturation [29, 30, 37]. Nevertheless, none of these adverse events occurred in the intracuff group.

### Sensitivity Analysis

To investigate the influence of a potential bias in our analysis, we conducted a sensitivity analysis. We attempted to exclude RCTs with (1) an unsatisfactory quality, such as inadequate blinding [11, 22, 23, 29, 30, 32, 34, 35], and per-protocol analysis [24, 32, 40]; (2) insufficient data disclosure regarding participant sex [31, 32, 36, 38], the surgical procedure [22, 30, 31, 33, 37, 39], and ETT size [10, 30, 37, 39]; (3) non-generalizable results, which have focused only on females [23, 24, 35], smokers [36, 39], and children [28]; (4) inconsistent anesthetic care, such as interference in continuous opioid infusion [12, 31, 32, 35, 36, 40], premedication [11, 12, 28, 30, 32, 34–36], ETT lubricant [10–12, 34, 36, 38], cuff prefilling [22, 30, 38], and filling strategy [31, 33, 39, 40], cuff pressure monitor [23, 24, 31–32, 38–40], as well as the avoidance of N_2_O [12, 24, 28, 31, 40], and inhalation anesthetics [31, 37]; and (5) the alternative conditions of intracuff lidocaine [29, 33]. None of these sensitivity analyses have influenced the primary outcomes (S2 Table).

## Discussion

This systematic review and meta-analysis demonstrates that intracuff lidocaine used in patients receiving ETGA is associated with significantly reduced incidence of POST, reduced POST severity as evaluated by pain scores at 1 and 24 h postoperatively, and lower risk of other postintubation emergence phenomena such as coughing, agitation, and dysphonia when compared with other interventions such as intracuff air or inflated saline. Both alkalinized and non-alkalinized lidocaine in the subgroup analyses showed significant benefits in emergence phenomena prevention compared with the control. No complications related to lidocaine overdose or endotracheal cuff rupture was reported.

One previous systematic review and meta-analysis examined the efficacy of prophylactic lidocaine for the prevention of POST caused by endotracheal intubation [19], and although it comprised 1232 patients from 15 studies, only 5 of them specifically evaluated the effects of intracuff administration without any subgroup analysis. In the present analysis, we included 14 additional trials, and not only reviewed the incidence and severity of POST but also postintubation-related emergence phenomena and conducted an extensive sensitivity analysis to achieve a high level of robustness. Our analysis determined that intracuff lidocaine is effective in preventing and alleviating POST as well as coughing, agitation, hoarseness, and dysphonia during emergence.

Intracuff inflation is an optimal route for lidocaine administration to prevent emergence phenomenon. First, unlike the inconclusive benefit of external topical application as tube lubricant [16, 38], our result is definite. Moreover, evidence shows that mucosa damage and cuff rupture might be associated with lidocaine gel or spray on the ETC [41, 42]. Second, intracuff administration prevents the risk of prolonged sedation after intravenous lidocaine injection [18, 43]. Third, inflating ETC with lidocaine could avoid the cuff overinflation due to rapid trans-cuff N_2_O diffusion during general anesthesia [38, 44, 45]. Since overinflated cuff might bring forth damage to pharyngeal mucosa and recurrent laryngeal nerve palsy [46], using liquid to replace air as cuff medium should be carefully considered [47, 48].

Our results revealed that intracuff alkalinized lidocaine provided an improved preventive effect compared with non-alkalinized lidocaine. To achieve a significant therapeutic effect, large doses of lidocaine (200 to 500 mg) might be required if it was not alkalinized [20, 22, 29, 32, 49]. Estebe et al. reported that alkalinized lidocaine diffused through the membrane of cuff 60 times more than non-alkalinized one in a 6-h period. Thus, a low dose lidocaine (40 mg) could offer adequate protection after alkalinization [10]. As for the plasma lidocaine level in different route, intravenous lidocaine may reach 2 to 3 μg/mL [50, 51], topical application was ranged from 0.43 to 1.5 μg/mL [52], whereas alkalinized lidocaine yielded below 0.08μg/mL [11, 21]. This indicated that intracuff alkalinized lidocaine inflation was attributable to a local effect, rather than to a systemic one. However, the most appropriate dosage and pH value for intracuff inflation may need further evaluation.

Age and surgical type also should be considered. In our review, one study evaluated children (aged 6 to 12 years) for dental surgery with N_2_O free ETGA, and the intracuff alkalinized lidocaine group experienced a significant reduction in the incidence and severity of coughing during extubation and POST [28]. However, a significant prolongation to spontaneous ventilation occurred before extubation in the intracuff alkalinized lidocaine group compared with the control group (16.4 ± 3.1 min and 9.4 ± 1.7 min, respectively). Another study with adult patients undergoing thyroidectomy was also free of N_2_O for patients undergoing ETGA [12]. Compared with the air group, the intracuff alkalinized lidocaine groups experienced a significant reduction in the soreness of the throat at 24-h postoperation. Further investigations on intracuff alkalinized lidocaine could focus on these special populations or head and neck surgery.

The significant heterogeneity among RCTs we selected is attributed to various factors. First, the characteristics of the participants varied. There are studies where only female patients or smokers were included, and in other studies didn’t even show the sex percentage of the participants. Second, various types of surgery were considered in this study. Third, although we focused on intracuff lidocaine, the concentration of lidocaine with or without alkalinization varied among studies. Fourth, the anesthetic interventions were relatively varied in the included studies. From the different ETT sizes and the techniques of maintenance of anesthesia (eg, the use of N_2_O, continuous opioid infusion, inhalation gases, or both), these differences exaggerated the heterogeneity of this study.

We performed a thorough search of clinicaltrial.gov. Four trials had investigated ETGA patients with intracuff lidocaine for POST, coughing, or the emergence phenomenon. Two studies were still recruiting participants. Two studies were complete, in which one study had published the initial data online. We look forward to further publications to investigate the effect of intracuff lidocaine.

Our research had a few limitations. First, the sample sizes in certain RCTs were relatively small. However, in this review, by using a comprehensive search for eligible studies, with no language limitations, systematic and explicit application of eligibility criteria, and a careful consideration of the study quality, as well as a rigorous analytical approach, we showed these might compensate for the above limitations. Second, the variability of the clinical factors and the non-uniform reporting of clinical parameters contributed to the observed heterogeneity, and most of the studies have not reported the details of the generation and concealment of allocation, and the clear definition of POST, which could have resulted in a potential bias. Because the participants included in the studies underwent different surgeries and were subject to various anesthesia strategies, we performed an extensive sensitivity analysis to make an a priori determination, which enabled us to assess sources of heterogeneity when present, and also to identify the sub-grouping of patients that could potentially benefit from this method. Third, several of our secondary outcomes were variably reported, and not all planned sensitivity analyses could be performed because of insufficient data. Fourth, although the subgroup analyses showed that the alkalinized lidocaine provided better performance than their non-alkalinized counterparts when compared with the control, the therapeutic effects between groups still needed to be verified by direct comparison. Last but not least, the publication bias may overestimate the efficacy of intervention and is the most vital threat to the validity of this meta-analysis.

In conclusion, our meta-analysis ascertained the effectiveness of intracuff lidocaine used in the prevention of emergence phenomenon. There was no report of lidocaine overdoses or systemic toxicity or of endotracheal cuff rupture in any of these studies. Further RCTs are required to overcome the limitations of heterogeneity as well as to determine the optimal dosage and application modalities of intracuff lidocaine to prevent the postintubation emergence phenomenon.

## Supporting Information

S1 PRISMA Checklist PRISMA 2009 Checklist to be included with meta-analyses. (DOCX)

S1 Fig Funnel plot of the lidocaine and control groups, showing the incidence of POST at 1 h. (EPS)

S1 Table Details of the search strategy. (DOCX)

S2 Table Sensitivity analyses: The effect of potential biases on primary outcomes. (DOCX)
