# Supplementary material for: Effect of Intracuff Lidocaine on Postoperative Sore Throat and the Emergence Phenomenon: A Systematic Review and Meta-Analysis of Randomized Controlled Trials
Source: PLoS One. 2015 Aug 19;10(8):e0136184. doi: 10.1371/journal.pone.0136184 (PMC4544846; doi:10.1371/journal.pone.0136184)
Supplement: S1 Table — (DOCX) [file pone.0136184.s003.docx]

S1 Table. Details of the search strategy

| Search strategy terms for PubMed, EMBASE, and Cochrane databases completed on September 10, 2014. |
| --- |
| The PubMed search terms were as follows:  ((((lidocaine) OR ligocaine) OR xylocaine)) AND (((((((((((emergence phenomenon) OR sore throat) OR coughing) OR buckling) OR dysphagia) OR dysphonia) OR difficult swallowing) OR laryngospasm) OR bronchospasm) OR cyanosis) OR desaturation) |
| The Cochrane search terms were as follows:  ID Search  #1 MeSH descriptor: [Lidocaine] explode all trees  #2 MeSH descriptor: [Pharyngitis] explode all trees  #3 MeSH descriptor: [Deglutition Disorders] explode all trees  #4 MeSH descriptor: [Cough] explode all trees  #5 MeSH descriptor: [Dysphonia] explode all trees  #6 MeSH descriptor: [Laryngismus] explode all trees  #7 MeSH descriptor: [Bronchial Spasm] explode all trees  #8 MeSH descriptor: [Cyanosis] explode all trees  #9 #2 or #3 or #4 or #5 or #6 or #7 or #8  #10 #1 and #9 |
| The EMBASE search terms were as follows:  “(('sore throat'/exp or 'sore throat' or 'coughing'/exp or 'coughing' or 'dysphonia'/exp or 'dysphonia' or 'dysphagia'/exp or 'dysphagia' or 'bronchial spasm'/exp or 'bronchial spasm' or 'cyanosis'/exp or 'cyanosis') and ('lidocaine'/exp or 'lidocaine')) and ('general anesthesia'/exp or 'general anesthesia')” . |
