# Supplementary material for: Effect of Intracuff Lidocaine on Postoperative Sore Throat and the Emergence Phenomenon: A Systematic Review and Meta-Analysis of Randomized Controlled Trials
Source: PLoS One. 2015 Aug 19;10(8):e0136184. doi: 10.1371/journal.pone.0136184 (PMC4544846; doi:10.1371/journal.pone.0136184)
Supplement: S2 Table — (DOCX) [file pone.0136184.s004.docx]

**S2 Table. Sensitivity Analyses: The Effect of Potential Biases on Primary Outcomes**

| Potential Bias or Limitations Excluded | Incidence of POST, 1 h (RR, 95 % CI; *I*^2^) | Incidence of POST, 24 h (RR, 95 % CI; *I*^2^) | POST pain score (mm), 1 h (MD, 95 % CI; *I*^2^) | POST pain score (mm), 24 h (MD, 95 % CI; *I*^2^) | Incidence of coughing  (RR, 95 % CI; *I*^2^) |
| --- | --- | --- | --- | --- | --- |
| **Overall** | 0.46, 0.31 to 0.68; 75 % | 0.41, 0.25 to 0.66; 59 % | -16.43, -21.48 to -11.38; 72 % | -10.22, -13.5 to -6.94; 70 % | 0.43, 0.31 to 0.62; 85 % |
| **RCT quality** |  |  |  |  |  |
| Single blinded^11,22,29,30,32,34,35^ | 0.48, 0.28 to 0.82; 81 % | 0.41, 0.19 to 89; 66 % | -15.23, -22.12 to -8.34; 77 % | -8.38, -12.1 to -4.66; 56 % | 0.53, 0.37 to 0.74; 83 % |
| No blinding^23^ | 0.41, 0.26 to 0.65; 77 % | 0.35, 0.22 to 0.56; 42 % | -17.88, -22.64 to -13.12; 66 % | -10.9, -14.18 to -7.62; 69 % | 0.43, 0.29 to 0.62; 87 % |
| Per-protocol^24,32,40^ | N/A | N/A | N/A | N/A | 0.41, 0.27 to 0.62; 88 % |
| **Participants** |  |  |  |  |  |
| No gender reported^31,32,36,38^ | 0.57, 0.4 to 0.8; 68 % | 0.49, 0.31 to 0.78; 58 % | N/A | N/A | 0.44, 0.3 to 0.65; 87 % |
| No surgery reported^22,30,31,33,37,39^ | 0.36, 0.22 to 0.58; 58 % | 0.28, 0.11 to 0.72; 69 % | -18.12, -23.67 to -12.58; 72 % | -10.3, -14.1 to -6.49; 77 % | N/A |
| Surgical site at neck^12^ | N/A | N/A | -16.41, -22.31 to -10.51; 75 % | -9.71, -13.42 to -6; 73 % | 0.47, 0.34 to 0.66; 83 % |
| Female only^23,24,35^ | 0.42, 0.27 to 0.67; 79 % | 0.38, 0.24 to 0.61; 40 % | -17.88, -22.64 to -13.12; 66 % | -10.9, -14.18 to -7.62; 69 % | 0.39, 0.26 to 0.59; 87 % |
| Smoker only^36,39^ | 0.47, 0.32 to 0.70; 76 % | 0.42, 0.25 to 0.68; 61 % | N/A | N/A | 0.44, 0.31 to 0.64; 86 % |
| Children only^28^ | 0.47, 0.32 to 0.71; 76 % | 0.43, 0.26 to 0.7; 59 % | -16.49, -21.92 to -11.06; 75 % | -10.58, -14.13 to -7.03; 72 % | 0.42, 0.29 to 0.62; 87 % |
| **Anesthetic use** |  |  |  |  |  |
| No N_2_O use^12,24,28,31,40^ | 0.43, 0.27 to 0.67; 74 % | 0.43, 0.26 to 0.7; 59 % | -17.58, -24.39 to -10.77; 79 % | -10.27, -14.81 to -5.73; 79 % | 0.4, 0.25 to 0.65; 87 % |
| No inhalation gas use^31,37^ | 0.41, 0.27 to 0.64; 73 % | 0.33, 0.17 to 0.63; 63 % | -17.23, -22.48 to -11.99; 72 % | -10.39, -13.93 to -6.86; 73 % | 0.39, 0.25 to 0.6; 88 % |
| Continuous opioid^12,31,32,35,36,40^ | 0.44, 0.28 to 0.69; 76 % | 0.46, 0.28 to 0.75; 61 % | -17.37, -23.55 to -11.18; 75 % | -9.86, -13.92 to -5.8; 76 % | 0.47, 0.32 to 0.71; 86 % |
| Premedication^11,12,28,30,32,34-36^ | 0.56, 0.37 to 0.85; 80 % | 0.56, 0.33 to 0.94; 66 % | -16.04, -23.62 to -8.46; 82 % | -8.68, -11.74 to -5.61; 44 % | 0.57, 0.4 to 0.82; 82 % |
| **ETT/cuff choice** |  |  |  |  |  |
| No size reported^10,30,37,39^ | 0.48, 0.32 to 0.71; 76 % | 0.36, 0.19 to 0.7; 63 % | -14.67, -19.81 to -9.53; 59 % | -10.88, -14.81 to -6.95; 69 % | 0.39, 0.25 to 0.59; 81 % |
| ETT lubricant use[^1^](#_ENREF_13)^0-12,34,36,38^ | 0.57, 0.4 to 0.81; 68 % | 0.45, 0.26 to 0.76; 65 % | -13.26, -21.26 to -5.26; 71 % | -9.16, -11.44 to -6.88; 0 % | 0.55, 0.39 to 0.76; 67 % |
| PREFILL^20,30,38^ | 0.53, 0.35 to 0.8; 72 % | 0.42, 0.23 to 0.78; 62 % | -17.11, -22.48 to -11.75; 72 % | -10.12, -13.63 to -6.61; 74 % | N/A |
| No MLT^31,33,39,40^ | 0.37, 0.23 to 0.59; 56 % | N/A | -17.23, -22.48 to -11.99; 72 % | -10.39, -13.93 to -6.86; 73 % | 0.42, 0.28 to 0.62; 86 % |
| No pressure monitor^23,24,31-32,38-40^ | 0.42, 0.26 to 0.67; 68 % | 0.39, 0.25 to 0.6; 36 % | -18.95, -23.72 to -14.18; 63 % | -11.17, -14.71 to -7.63; 73 % | 0.36, 0.21 to 0.6; 90 % |
| **Lidocaine** |  |  |  |  |  |
| High concentration^29^ | 0.46, 0.3 to 0.71; 77 % | 0.45, 0.27 to 0.73; 56 % | -15.22, -20.58 to -9.87; 71 % | -10.04, -14.17 to -5.91; 73 % | 0.45, 0.32 to 0.65; 88 % |
| High temperature^33^ | 0.41, 0.26 to 0.66; 71 % | N/A | N/A | N/A | N/A |

CI = confidence interval; ETT = endotracheal tube; MLT = minimal leakage technique; MD = mean difference; N/A = not applicable; RR = relative risk; POST = postoperative sore-thorat; PREFILL = cuff injected with lidocaine 90 min before intubation.
